# Prevalence and associations of depression among patients with cardiac diseases in a public health institute in Trinidad and Tobago

**DOI:** 10.1186/s12888-018-1977-3

**Published:** 2019-01-07

**Authors:** Mandreker Bahall

**Affiliations:** School of Medicine and Arthur Lok Jack Graduate School of Business, University of the West Indies, St. Augustine, House #57 LP 62, Calcutta Road Number 3, McBean, Couva Trinidad, Trinidad and Tobago

**Keywords:** CVD risk factors, Depression, Prevalence of depression, Associations of depression, PHQ-9

## Abstract

**Background:**

Psychosocial issues are major determinants as well as consequences of cardiovascular disease (CVD). This study sought to assess the prevalence and identify factors associated with depression among patients with cardiac disease in a public health institute in Trinidad and Tobago.

**Methods:**

A cross-sectional study was conducted with a convenience sample of 388 hospitalised, stable, adult patients with cardiac disease admitted in the only tertiary public health institute in South Trinidad. Patients were identified and interviewed 3 to 5 days after admission using a questionnaire comprising questions on demographic, medical, and lifestyle issues and the 9-item Patient Health Questionnaire (PHQ-9).

**Results:**

The prevalence of clinically significant depression (PHQ-9 > 9) among hospitalised patients with cardiac disease was 40.0%. However, the prevalence of non-minimal depression (PHQ-9 ≥ 5) in this study was 78.4%. It was greater among women (83.1%) than among men (72.9%). Non-minimal depression was associated with sex (*p* = 0.015), employment status (*p* = 0.007), hypertension (*p* = 0.017), stressful life (*p* ≤ 0.001), feelings of depression (p ≤ 0.001), regular exercise (p ≤ 0.001), and living alone (*p* = 0.006). Age, ethnicity, education level, income, or religious affiliations were not associated (*p* > 0.05) with depression. Participants diagnosed with depression commonly reported feeling tired (81.2%), having trouble sleeping (74.7%), and moving/speaking slowly (73.5%). Patients with self-claimed depression (past or current) were four times more likely to have depression and those with self-reported stress and loneliness were twice as likely to have depression. Employed patients and those who exercised regularly were approximately 50% less likely to have depression.

**Conclusions:**

Clinical depression prevalence among hospitalised patients with cardiac disease was 40.0%. Approximately twice as many (78.4%) had non-minimal depression, with higher prevalence among women. Employment, sex, hypertension, stressful life, feelings of depression, regular exercise, and living alone were associated with non-minimal depression. Patients with self-claimed depression, stress, and those living alone had a much higher likelihood of having depression, while those who were employed and exercised regularly were approximately half as likely to have depression.

## Introduction

Many patients with cardiac disease feel anxious, worried, and depressed. Depression, defined as a mood disorder that causes a persistent feeling of sadness and loss of interest [1], has moved from the fourth leading cause of disability worldwide in 1996 [2] to the leading cause of disability globally in 2017 according to the World Health Organization [3]. Cardiac disease treatment has nonetheless focused on physical symptoms, such as angina, arrhythmias, and heart failure, and not on psychological complications [4]. Depression can lead to psychological, physical, and social consequences [5]. Psychological effects include anxiety, fear, sadness [6], hopelessness [6], guilt [6], and irritability [6]. Social consequences involve a change in the functionality of a person and may include substance use and abuse [7], social withdrawal [7], and decreased performance in daily activities [7]. The long-term effects of depression have been linked to brain damage [8], negative impact on the heart [9], and reduced physical activity or the development of sedentary lifestyles [10]. Depression influences lifestyle in areas such as smoking, eating, exercising, adjustment to family and social life, and employment [11, 12]. It is associated with an increase in hospital readmissions [13], increased incidence of heart failure [14], double the long-term risk of death after a heart attack [15], increased mortality risk by twofold [5, 16], and decreased quality of life and increased medical morbidity [17]. Depression is also associated with elevated risk of cardiovascular (CVD) [18] and coronary artery disease (CAD). [19]

Accompanying depression on patients with cardiac disease can be more pronounced because of accompanying medical and social factors. Medical factors can be human immune deficiency virus (HIV), cancer, and end-stage renal diseases. Socio-economic status, particularly education and income, may be associated with depression. [20] In addition to these effects, murder (463 recorded in 2016) [21], sexual offences (693 in 2014) [22], rape (159 recorded in 2014) [22], suicide (14.5 per 100,000 in 2015) [23], divorce (2814 in 2015) [24], and unemployment [25] may have a considerable impact on depression. Depression in patients with coronary heart disease (CHD) has a high persistence rate if left untreated [26] and can worsen CVD burden, which is already the leading cause of mortality in Trinidad and Tobago [27] in line with the worldwide data [28].

Studies conducted in Trinidad and Tobago in selected cross-sectional samples have revealed the prevalence of depression to be 12.8% among adults visiting family physicians [29], 28.3% among patients with chronic diseases [30], 14.0% among adolescents [31], and 17.9% among patients treated for type 2 diabetes mellitus [32]. However, studies on the prevalence of depression among patients with cardiac diseases have not been reported. This study aimed to determine the prevalence and identify factors associated with depression among patients with cardiac diseases admitted for cardiac care to public hospitals in Trinidad and Tobago.

## Methods

The target population consisted of all patients admitted to public hospitals in Trinidad and Tobago for cardiac care. The sampled population however consisted of all patients admitted for cardiac diseases at the San Fernando General Hospital. The San Fernando General Hospital is one of the four hospitals providing tertiary care in Trinidad and Tobago. It is a public, 745-bed facility that serves half the population of Trinidad or approximately 600,000 people. Medical admissions account for a total of approximately 1400 each month [33] of which one-fifth pertains to cardiac disease, mainly unstable angina (IHD), arrhythmias, heart failure, or valvular heart disease. A single institution was selected because of cost considerations, similarity in the conditions among hospitalised patients with cardiac disease, and the large number of patients this particular hospital treats.

### Selection of participants

During the 5-month period from November 1, 2015 to March 31, 2016, all patients admitted to the hospital for cardiac diseases were examined for eligibility for participation in the study. The medical ward admission books were used to identify patients with a cardiac diagnosis. The identified patients with cardiac disease were approached for discussion on the nature of the study and their willingness to grant consent for participation. The eligibility criteria were stable cardiac disease for a minimum of 3 months, age 18 years or older, absence of confusion (ability to understand, think clearly, and produce meaningful understandable statements), and ability to communicate in the native English language for approximately 20 min and recall experiences without difficulty. The exclusion criteria were severe cardiac illness (i.e. patients who are unstable, very short of breath, or exhausted), and accompanying major debilitating comorbidities such as dialysis, terminal stage of cancer (cancer that has spread to various parts of the body), acquired immune deficiency syndrome (AIDS) (i.e. patients who were symptomatic: weak, emaciated, and likely to have opportunistic infections), or stage 1 V heart failure (i.e. patients who were very symptomatic at rest or minimal exertion). Medical students acted as research assistants and were trained on the identification of patients with cardiac disease, the conduction of interviews, and the collection of data. Through convenience sampling, patients with cardiac disease, identified from the patient records and whose diagnoses were confirmed by the treating physicians, were selected for possible participation in the study. Potential participants were briefed on the nature of study, and their willingness to participate was ascertained. They were also informed of their rights (freedom of choice to participate, discontinue, or refuse to participate). Patients whose verbal consent was acquired were interviewed. Interviews were stopped and resumed if the interviewees were in the meantime needed for medical examinations, consultations, or investigations. Face to face interviews were conducted at the patient’s bedside 3 to 5 days after admission. This would allow time for patient stabilisation and would avoid interference with medical interventions. Since the average length of stay for medical patients, including those with cardiac diseases, is 5.6 days, most patients were available for interview 3 days after admission. A sample size of 388 was determined to be sufficient using a 5% margin of error and a prevalence of 50% in the population.

### Data collection instrument

The data collection instrument was a questionnaire that contained survey items related to patient demographics and a depression diagnostic tool. Data collected included socio-demographic variables (age, sex, height, weight, ethnicity, level of education (primary, secondary, or tertiary), current employment status, and religion), self-reported medical and lifestyle history (hypercholesterolemia, current smoking (i.e. smoking up to within 30 days of the interview), diabetes mellitus, hypertension, abdominal obesity, stressful life, depression, daily consumption of substantial fruits and vegetables, exercise (at least 3 times per week for 20 min), regular alcohol consumption, and family history of ischaemic heart disease (female parent or siblings younger than 65 years old and male parent or siblings younger than 55 years old reported to have or treated for heart disease)), other medical history (chronic obstructive pulmonary disease, cancer, end-stage renal disease, peripheral vascular disease, stroke/transient ischaemic attack, and psychosis), specialised examinations/treatment (angiography, angioplasty, and coronary artery bypass grafting), and others such as social support (source of support and care). Patients were guided on the interpretation of social support (receiving the required help in daily chores), of exercise (exercising regularly for 20 min at least 3 times per week), feeling stressed – anxious, irritable, and depressed – ‘down’, sad, or dejected. The type and duration of cardiac disease were also recorded.

The PHQ-9 was selected to measure depression among the commonly used instruments. The PHQ-9 has been widely used among patients with cardiac disease with good sensitivity and specificity and it is easy to administer. A cut-off score of ≥5 for minor depression had sensitivity of 0.91 and specificity of 0.81 for a Sri Lankan population [34]. The PHQ-9 has a sensitivity of 54% and specificity of 90% for scores ≥10 in a United States and Canada population [35]. Overall, the PHQ-9 was reported to have 94% sensitivity and 84% specificity for scores > 8 and achieved greater accuracy than the depression component of the Hospital Anxiety and Depression score (cut-off of 5, sensitivity = 81%; specificity = 77%) [36]. The PHQ-9 comprises nine questions: ‘little interest or pleasure in doing things’, ‘feeling down, depressed, or hopeless’, ‘trouble falling or staying asleep or sleeping too much’, ‘feeling tired or having little energy’, ‘poor appetite or overeating’, ‘feeling bad about yourself or that you are a failure or have let yourself or your family down’, ‘trouble concentrating on things such as reading the newspaper or watching television’, ‘moving or speaking so slowly that other people could have noticed or the opposite – being so fidgety or restless that you have been moving around a lot more than usual’, and ‘thoughts that you would be better off dead or of hurting yourself in some way’. The questions are scored using a scale of 0 to 3: not at all (0), several days (1), more than half the days (2), and nearly every day (3). The PHQ-9 scores classify depression as mild (score: 5–9), moderate (10–14), moderately severe (15–19), and severe (20–27) [37]. While most studies have used a score signifying higher than moderate depression as warranting intervention, even mild depression should be identified because of its role in worsening CAD. A study with hospitalised patients with cardiac disease utilised depression with a cut-off of 5 or non-minimal depression (PHQ-9 ≥ 5) [38] to determine depression prevalence. In this study, tests of association and strength of association were conducted utilising PHQ-9 ≥ 5. For this, depression scores were dichotomised into less than mild depression (PHQ-9 < 5) or depression (PHQ-9 ≥ 5). In addition, a significant clinical depression score (PHQ-9 > 9) was calculated to compare the prevalence among various studies, since most of these used a cut-off for depression of PHQ-9 > 9.

### Statistical analysis

Data collected were entered and stored in a password-secured computer. SPSS, Version 21 (IBM Corp., Armonk, NY) was used to analyse the data through both descriptive and inferential statistical methods. Descriptive methods included frequency and percentage distributions tables, bar graphs, and summary statistics. Inferential methods included forming 95% confidence intervals (CIs), and chi-square tests of association as was required for this cross-sectional study. Odds ratios as opposed to relative risks were used to determine the strength of the associations, since the cross-sectional sample had a high prevalence of depression [39].

Ethical approval was granted by the ethics committee of the South West Regional Authority on October 21, 2015.

## Results

**Fig. 1:**
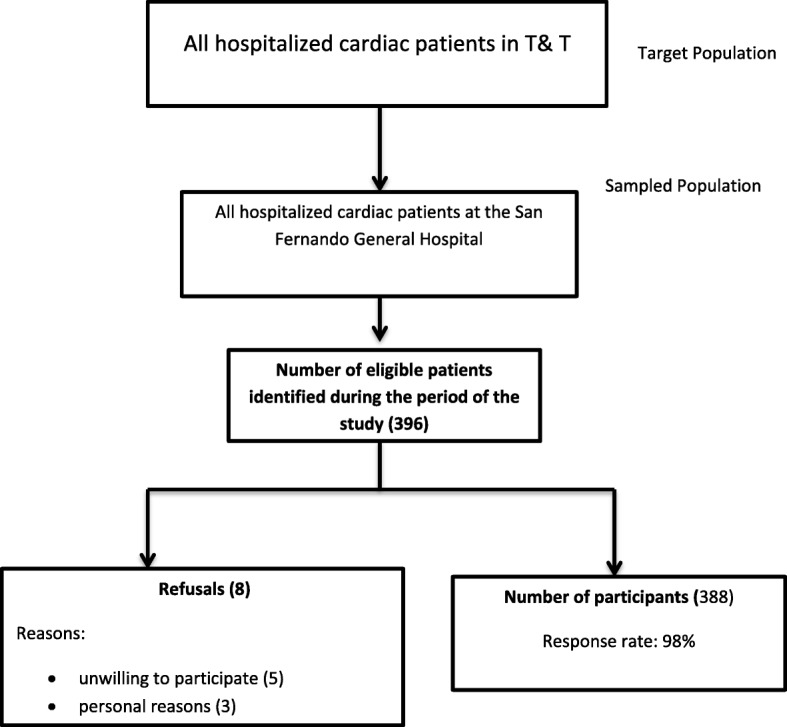
Selection of study participants

**Fig. 2:**
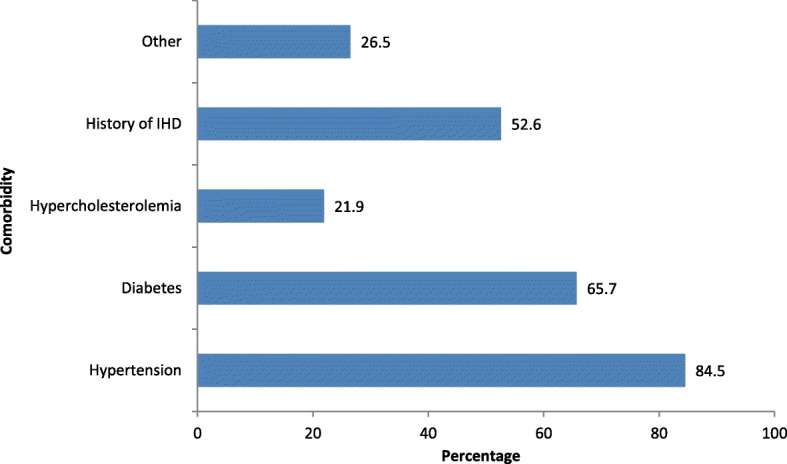
Patients’ cardiovascular disease comorbidities

**Fig. 3:**
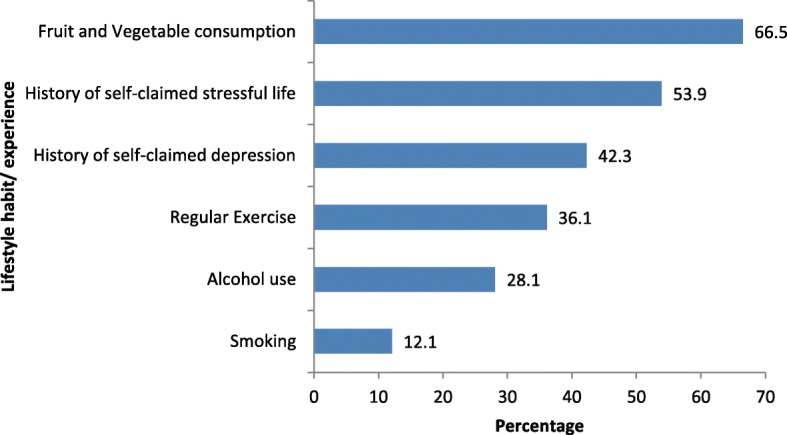
Lifestyle habits and experiences of cardiovascular disease

Of the 396 patients identified as being eligible for participation in the study during the period from November 1, 2015 to March 31, 2016, 388 (98.0%) provided verbal consent and eight (2.0%) refused (Fig. 1). The reliability (Cronbach’s alpha) of the questionnaire was 0.747 and the reliability (Cronbach’s alpha) of the PHQ-9 was 0.749, which exceed the minimum acceptable of 0.70 [40]. Patients were predominantly female (*n* = 207; 53.4%), aged 45–74 years (*n* = 268; 69.1%), of Indo-Trinidadian descent (*n* = 280; 72.2%), had at most a primary school education (*n* = 232; 59.8%), and had a monthly income of less than TT$5000 (*n* = 232; 59.8%). Table 1. The highest prevalent comorbidity was hypertension (84.5%) followed by diabetes (65.7%) (Fig. 2). The most common cardiac disease was ischaemic heart disease or coronary heart disease (75%), followed by cardiomyopathy (7.2%), valvular heart disease (3.1%), cardiac arrhythmia (4.1%) and heart failure (4.6%). Approximately half (53.9%) of the patients reported a history of self-claimed stressful life prior to diagnosis and a high percentage (42.3%) also had a history of self-claimed depression (Fig. 3). Smoking and alcohol use were not common among participants.

**Table 1:** Frequency distribution of socio-demographic variables

Variable	n	%
Sex		
Male	181	46.6
Female	207	53.4
Age group		
< 35	11	2.8
35–44	24	6.2
45–54	50	12.9
55–64	97	25.0
65–74	121	31.2
75–84	66	17.0
85 & over	19	4.9
Ethnicity		
Indo-Trinidadian	280	72.2
Afro-Trinidadian	83	21.4
Mixed	25	6.4
Education		
Less than primary	34	8.8
Up to Primary	232	59.8
Secondary	99	25.5
Tertiary	23	5.9
Monthly income $TT)		
< 25,000	70	18
2501–5000	232	59.8
5001–10,000	26	6.7
Over 10,000	11	2.8
Religion		
Islam	25	6.4
Hindu	157	40.5
Christian	201	51.8
Other	5	1.3
Religiosity		
Extremely religious	42	10.8
Very religious	217	55.9
Somewhat religious	112	28.9
Not religious	17	4.4

The prevalence of each of the nine PHQ-9 depression scale indicators was: feeling tired (88.5%), having trouble sleeping (85.6%), moving/speaking slowly (83.9%), feeling down, depressed, hopeless (70.5%), poor appetite/overeating (66.8%), trouble concentrating (61.8%), little interest/pleasure in doing anything (56,6%), feeling bad about self/failure/let family down (42.1%), and thoughts of being better off dead/self-harm (14.1%).

**Fig. 4:**
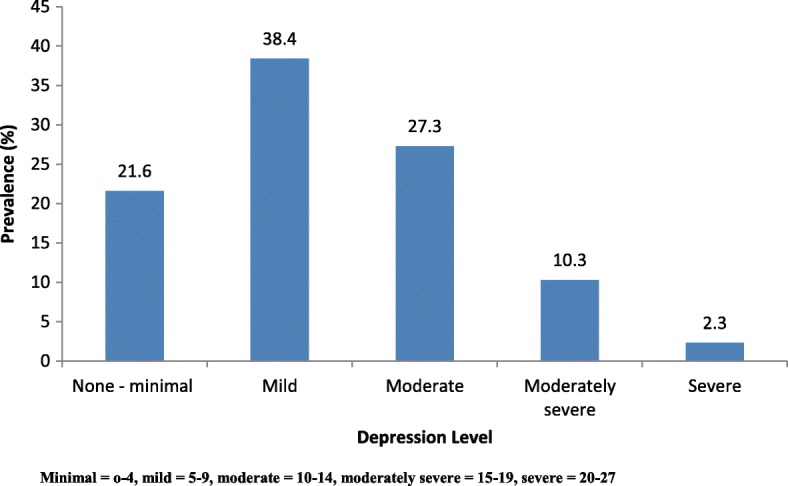
Patients’ Patient Health Questionnaire-9 depression category distribution

Depression prevalence or significant clinical depression based on PHQ > 9, i.e. at least moderate depression, was 40.2%. However, further analyses used depression based on non-minimal depression (PHQ-9 score ≥ 5), which was present in 78.4% [*n* = 304; 95% CI (73.9, 82.3)] (Fig. 4). Non-minimal depression (PHQ-9 score ≥ 5) was associated with a number of demographic and lifestyle variables and comorbidities such as sex (*p* = 0.015), employment (*p* = 0.007), hypertension (*p* = 0.017), previous stressful life (*p* ≤ 0.001), current stressful life (*p* = 0.001), previous feelings of depression (p ≤ 0.001), current feelings of depression (p ≤ 0.001), regular exercise (p ≤ 0.001), and loneliness (p ≤ 0.001). However, depression was independent of ethnicity (*p* = 0.090), education level (*p* = 0.209), religion (*p* = 0.689), and diabetes mellitus (*p* = 0.061).

Table 2 presents the odds ratios of non-minimal depression for significantly associated comorbidities, psychological, lifestyle, and CVD complications, measured with the associated 95% CIs, and the corresponding *p*-values. The participants of male sex [OR: 0.548; *p =* 0.015; CI (0.336, 0.894)], those who were employed [OR: 0.452; *p =* 0.007; CI (0.252, 0.811), and those who exercised at least three times per week [OR: 0.394; *p* = 0.001**;** CI (0.241, 0.645)] were approximately 50% less likely to be depressed. However patients with hypertension [odds ratio (OR): 2.063; *p* = 0.017; 95% CI (1.129, 3.767)], current stressful lifestyle [OR: 2.943; *p* = 0.001; 95% CI (1.771, 4.892)], previous stressful lifestyle [OR: 2.327; *p* = 0.001; CI (1.417, 3.822)], current feelings of depression [OR: 6.724; *p* = 0.001; 95% CI (3.432, 13.175)], previous feelings of depression [OR: 5.517; *p* = 0.001; CI (2.815, 10.813)], and loneliness [OR: 2.992; *p* = 0.006; CI (1.316, 6.798)] were more likely to be depressed. (Table 2).

**Table 2:** Odds ratios of associated factors and depression

			95% Confidence Interval	
Factors	OR	*p*-value	Lower	Upper
Sex (ref Female)	.548	0.015	0.336	0.894
^a^Employment	.452	0.007	0.252	0.811
Comorbidity				
^a^Hypertension	2.063	0.017	1.129	3.767
Psychological				
^a^Previous stressful life	2.327	0.001	1.417	3.822
^a^Current stressful life	2.943	0.001	1.771	4.892
^a^Previous feelings of depression	5.517	0.001	2.815	10.813
^a^Current feelings of depression	6.724	0.001	3.432	13.175
^a^Living alone	2.992	0.006	1.316	6.798
Lifestyle				
^a^Exercise 3 times per week for > 20 min	0.394	0.001	0.241	0.645

*CVD* cardiovascular disease, *IHD* ischemic heart disease, *CABG* coronary artery bypass graft, *TIA* transient ischemic attack

^a^reference values: unemployment, no hypertension, no previous stressful life, no current stressful life, no previous feelings of depression, no current feelings of depression, not living alone, and not exercising 3 time per week for > 20 min

## Discussion

The prevalence of clinically significant depression, i.e. at least moderate depression (PHQ > 9), was 40.0% (*n* = 155) for patients with cardiac disease commonly due to IHD, arrhythmias, and valvular heart disease. Varying depression prevalence has been found in other studies: 45.1% among patients with IHD [41] and 22% among those with cardiovascular disease [42], 14% for valvular heart disease [42], and 29% for arrhythmia without structural heart disease [42]. Studies by the American Psychiatric Association reported high prevalence of depression in patients with CAD [43]. Based on the PHQ-9, Haddad et al. found that 13.6% of patients with CHD (including those with a history of myocardial infarction (MI), angina, or those that had undergone coronary artery revascularisation procedures) were depressed [36]. Another study with patients with post-acute MI using the PHQ-9 found that 18.7% of patients met the PHQ-9 criteria for depression [44]. Polikandrioti et al. found that 17.4 and 24.2% of hospitalised patients with heart failure had minor and major depression, respectively [45]. Such variations may result from the differences in the composition of the samples in these studies or accompanying confounding factors. Depression prevalence was found to be higher among younger women [46, 47] and in patients with post-acute MI aged 65 years and older [48]. In the case of Trinidad and Tobago clinical depression may be high because of other confounding factors such as accompanying comorbid conditions and social determinants such as crime, disputes, traffic congestion, and economic issues.

The most common depressive symptoms experienced by patients in the last 2 weeks as reported in our study were feeling tired/having little energy (88.5%), moving or speaking slowly/fidgety, restless (83.8%), trouble falling asleep/staying asleep/too much sleep (83.5%), and feeling down, depressed, and hopeless (70.3%), in line with the results reported by Rohyans et al. who found that the most frequently reported depressive symptom by the patients on a scale from 0 (not at all) to 3 (nearly every day) was “feeling tired/no energy” with 69% of patients reporting a score of 3 (mean = 2.6) [49]. The next most frequently reported depressive symptom according to Rohyans et al. was “trouble falling or staying asleep” with 43% of patients reporting a score of 3 (mean = 2.0) [49]. Whooley et al. revealed depressive symptoms in 19.6% of patients with CVD [50]. However, among patients with CAD, the prevalence of major depressive disorder or experiences of an elevation in depressive symptoms was 20–40% [51]. The specific symptoms experienced by patients after admission may be related to the consequential effects of the cardiac condition [52]. In fact, mild depression may be a reflection of the somatic items on the PHQ-9. This somatic effect may be dampened, however, a few days after admission when the patient’s physical condition has improved. In addition, patients reported on their experience in the 2 weeks prior to interview.

In this study, the commonest risk factors were hypertension followed by diabetes mellitus, stressful life, and family history of IHD, with clinical depression ranking fifth. Furthermore, diabetes, hypertension, hypercholesterolemia, abdominal obesity, end-stage renal disease, cancer, and chronic obstructive pulmonary disease accompanying a cardiac problem were associated with increased levels of depression compared to the absence of these conditions. Multiple chronic conditions such as diabetes, heart disease, and arthritis are associated with significantly higher levels of depressive symptoms [53] as reported by other studies. Higher levels of depression were also found in patients with more health conditions [54] and those with chronic diseases and other non-cardiac medical illnesses [55].

Apart from clinical depression discussed above, the study also analysed the effect of non-minimal depression (PHQ-9 ≥ 5), which had a prevalence of 78.4% (*n* = 304). Although most studies define depression as moderate to severe depression, it may be useful to also examine non-minimal depression because, when ignored, it may have negative consequences. These patients may worsen psychologically and physically. Furthermore, mild depression may benefit from some form of intervention or monitoring.

### Associations and predictors

Age showed no association with non-minimal depression (PHQ-9 ≥ 5) in this study. This study also found no significant association with diabetes mellitus and non-minimal depression. This was unexpected. Increased depression among diabetics was identified by Anderson et al. who found that in controlled studies, the odds of depression in the diabetic group were twice as high as those in the non-diabetic comparison group (OR = 2.0, 95% CI 1.8–2.2) [56]. Al-Ghamdi et al. also concluded that depression is more common among diabetics (34%) than among non-diabetics (13%, *p* < 0.001) [57].

In this study, patients who had undergone open heart surgery intervention experienced more depression (83.3%, *p* = 0.49), than did those who had undergone angioplasty (72.4%, *p* = 0.42), both of which were not significantly associated with depression (Table 2). This contradicts the findings of Chaudhury et al. who reported that before and after receiving percutaneous transluminal coronary angioplasty, 32.1% and 3.6% of patients respectfully had clinically significant depression [58]. This may relate to other interventions that may accompany surgical procedures such as counselling and cardiac rehabilitation. Cardiac rehabilitation in Trinidad and Tobago is absent in the public health institutes. Some studies have identified higher depression levels among patients who subsequently became unemployed [59] and those with income, relationship, and participation restrictions [60, 61]. The absence of association between depression and age, ethnicity, monthly income, or religious affiliation in this study is in line with findings of Munga who showed that age, sex, and marital and socioeconomic statuses did not significantly affect the development of depression in patients with cardiac diseases [62].

There was a high correlation between self-reported stress and the depression score obtained from the PHQ-9. Patients with self-reported depression were four times more likely to be depressed (PHQ-9 > 9). There may be a case for treating those that self-report feelings of depression without elaborate screening for depression. However, this method may neglect cases of an occult nature that may be in greater need of assistance, since many of these patients may even be suicidal [63] and unwilling to share their feelings. Depression among patients with self-reported stress was twice as common as among those without self-reported stress. These findings are corroborated by Schrader et al. who showed that self-reported history of depression, anxiety, or stress [64] were useful predictors of mild or moderate to severe level of depressive symptoms at hospitalisation.

Social support, while not robustly examined, did not significantly impact depression prevalence in this study. This may be related to the high percentage (at least 80%) of patients who had some form of support and is comparable to previous findings of 79.9% of patients receiving support from a spouse or relative [55]. Social support refers to the various types of support that people receive from others and is generally classified into two or three major categories: emotional, instrumental, and (sometimes) informational support [65]. Low social support infers that the emotional, instrumental, and informational assistance/help that people receive from others is lacking. Low social support is a robust risk factor for major depressive disorder [66]. Higher depression levels were also found in those living alone [67] or those who experienced social neglect at the work place [59]. Freidmann et al. found that depression and social isolation predicted mortality in heart failure patients [68].

A multitude of other factors such as those pertaining to personality may also be related to depression. Individuals with negative illness beliefs [69] and those with high neuroticism and low extraversion scores were found to be more vulnerable to depression [70] as was reported by a study conducted among older CHD patients. Living alone, alcohol abuse, perception of medical care as being a substantial economic burden, and health status were identified as predictors of developing depressive symptoms [71].

This study found that employment and exercising at least three times per week for 20 min provided additional protective benefits, since these patients were 50% less likely to develop depression compared to those who were unemployed or exercised less following MI [72]. In fact, regular exercise can be used as an instrument to reduce depression in patients after CAD events [73]. Similarly, another study reported increased levels of depression associated with adverse changes in employment after experiencing an MI [74].

### The way forward

In this study, four patients or 1% reported receiving formal help from a psychiatrist or psychologist. This is despite at least 40.0% having clinical depression (moderate, moderately severe, or severe depression) based on PHQ-9 > 9. A large percentage of untreated patients has been reported in other studies; Smolderen et al. found that 528 (69.6%) patients had untreated depression [44]. Because of the high incidence of disease complications and the increased mortality associated with depressed patients with cardiac disease [3, 5], patients with depression must be identified and treated in an appropriate manner e.g. with counselling, psychotherapy, and/or pharmacotherapy [75] to improve their quality of life.

### Limitations

This was a single-centre study with non-debilitated patients with cardiac disease with a relatively small convenience sample. Convenience sampling has a degree of bias, although efforts were made to select all eligible patients during the study period. The exclusion of patients with severe health illnesses has led to underestimation of the prevalence of depression among patients with cardiac disease, since those with high disease severity may be more likely to be depressed. Conversely, there is a potential to spuriously inflate somatic symptoms after admission and thus, artificially inflate depression prevalence. Interviewing patients 3–5 days after admission may exclude some patients that had been discharged by that time. However, with an average length of stay of 5.6 days, most patients were available for interview. This study primarily depended on recall, which can be difficult for many patients. Certain questions may not have been sufficient to provide an adequate evaluation of variables such as social support, stress, or depression, since self-reports were used in the study. Though patients were willing to share their feelings, many responses could have been exaggerated or under-reported. The population was mainly Indo/Afro Trinidadian of lower socioeconomic status who sought support in the public healthcare system. Although generalisation would be difficult, this study produced findings that could be extrapolated to similar populations. Psychosocial issues such as divorce, suicide, unemployment, and crime, which may influence the extent and effect of depression in patients with cardiac disease, have not been addressed. Although the research assistants would inform the attending physician of patients with suicidal ideation, the patients were not identified, and no formal report could be conducted.

## Conclusion

Depression was very common among patients with cardiac disease who presented most commonly with feelings of tiredness, insomnia, and inertia. Associated factors of depression included sex, employment, hypertension, previous and current stressful life, previous and current feelings of depression, living alone, and regular exercise. The OR for depression was the highest among patients who self-reported feeling stressed and depressed and among those living alone. Patients that regularly exercised and were employed were approximately 50% less likely to have depression. Screening of all patients with cardiac disease is essential to identify and treat the patients at greater risk of depression.

## Abbreviations

CAD: Coronary artery disease
CI: Confidence interval
CVD: Cardiovascular disease
IHD: Ischaemic heart disease
MI: Myocardial infarction
OR: Odds ratio
PHQ-9: Patient Health Questionnaire-9
